# Illuminating Insights into the Biodiversity of the Australian Psyllids (Hemiptera: Psylloidea) Collected Using Light Trapping

**DOI:** 10.3390/insects11060354

**Published:** 2020-06-05

**Authors:** Francesco Martoni, Gary S. Taylor, Mark J. Blacket

**Affiliations:** 1 Agriculture Victoria Research, AgriBio Centre, 5 Ring Road, Bundoora, VIC 3083, Australia; mark.blacket@agriculture.vic.gov.au; 2 Department of Ecology & Evolutionary Biology, School of Biological Sciences and Australian Centre for Evolutionary Biology & Biodiversity, The University of Adelaide, North Terrace, SA 5005, Australia; gary.taylor@adelaide.edu.au

**Keywords:** Sternorrhyncha, Aphalaridae, Phacopteronidae, Psyllidae, Triozidae, barcoding, COI

## Abstract

The superfamily Psylloidea includes numerous species which play a key role in Australian ecology and biodiversity, as well as pests and biological control agents, and sometimes threatened species of conservation concern. Different psyllid sampling and collection techniques are usually performed depending on the nature and aim of the study: from the beating and sweeping of psyllid host plants for conservation and biodiversity assessment, to suction and sticky traps in agriculture. Due to a general lack of information on its efficacy for psyllids, however, light trapping has not usually been employed. Here we present the results obtained trapping psyllids using different light sources and we discuss the strengths and weaknesses of this technique to assess psyllid biodiversity. In particular, we highlight the strength of using this methodology paired with DNA barcoding, to cast some light on psyllid biodiversity. The results obtained here suggest that the psyllid fauna of Australia is heavily understudied and the number of undescribed species might be many times higher than previously expected. Additionally, we report, for the first time, the species *Trioza adventicia* Tuthill 1952, and *Cryptoneossa triangula* Taylor 1990 in the state of Queensland.

## 1. Introduction

Psyllids, also known as jumping plant lice, belong to the superfamily Psylloidea (Hemiptera: Sternorrhyncha), grouped into more than 3800 species recognized worldwide [1]. Australia is a hot spot for psyllid diversity, with a fauna exceeding the 400 described species and a high number of species awaiting description [2,3]. As such, psyllid diversity in Australia provides important ecological functions, namely by providing food for a wide range of native birds [4]. The Australian psyllid fauna includes important pest species for forestry as the agents of eucalypt dieback, e.g., in the genera *Cardiaspina* [5,6] and *Glycaspis* [7], especially *G. brimblecombei* Moore, that has been accidentally introduced in non-native countries [8]. It also includes species of conservation significance, such as those co-threatened on threatened host plants, such as *Acizzia hughesae* Taylor and Moir, *A. mccarthyi* Taylor and Moir and *Trioza barrettae* Taylor and Moir [9,10]. For these reasons, only in the last decade have psyllid studies spanned from biodiversity assessments and regional checklists [11,12,13], to phylogenetic studies [14,15], to new taxonomic species descriptions [16,17,18,19,20], research on insect host-plant interactions [21] and to molecular and genomic analyses focusing on psyllids-bacteria interactions [22,23].

Independently of the focus of the study, the sampling and collection of psyllids is the first fundamental step, providing different arrays of information depending on the methodology used. Indeed, psyllids are collected using a variety of methods. For example, in agriculture and biosecurity, psyllids can be collected using sticky traps [24], suction traps, stem-tap traps or visual sampling [25], or even traps constructed using three-dimensional printer technology [26]. These traps, however, are not meant to provide information on the host plants for the psyllids collected, since they are used to assess the psyllids presence on certain monoculture crops of economic interest, such as citrus or potatoes [27]. When taxonomic studies or a biodiversity assessment require links to host plant associations, psyllids are swept using nets, or beaten onto trays to be then collected and preserved [16,28], or reared from plant foliage or galls [18]. However, due to the host specificity of a vast number of psyllid species, host plant-targeting collection mostly results in just a single, or few species of psyllids collected from each species of plant [21,29], which can make a biodiversity assessment extremely time consuming. Notwithstanding, some species of plant may be host to more than one species of psyllid [3,30].

While targeting mostly nocturnal insect species [31], light trapping can be used for the collection of a wide range of insect orders, including Diptera, Plecoptera and Hemiptera [32,33,34,35], in addition to the more common Lepidoptera [31]. Therefore, this method is widely considered as a useful way to sample insect biodiversity [33,34,35]. This is especially true in areas that are difficult to survey by other means, such as the tropical rainforest [36]. However, this method has proven to be very effective, even in urban areas [34,37]. Due to the nature of this collection method, the number of individuals trapped that belong to the same species might be variable, sometimes resulting in a single specimen collected per species [34]. 

Such variability in the number of individuals collected might complicate the process for their identification. The morphological identification of psyllids in Australia is a challenging task, for a number of reasons. Firstly, the keys available to genera [3] do not include a number of genera that have been described in the last 16 years, such as *Casuarinicola* [38], *Acanthocasuarina* [16] and *Myotrioza* [17], or that have arrived in the country since, such as *Bactericera* [39]. Secondly, in order to provide a precise species identification of psyllids, it is usually required to examine both sexes and/or to know the host plant, which might not be possible using light traps. Finally, even when a sufficient number of specimens is collected, this might belong to one of the many undescribed taxa for which species-level identification is not yet possible [2,40].

Since the paucity of specimens might complicate morphological identification, DNA barcode data, comprising a short DNA sequence from the 5′ region of the subunit I of the cytochrome oxidase c (COI) gene [41], could be matched on a publicly available database, such as BOLD [42], as an alternative means of identification. Furthermore, DNA barcoding has proved extremely valuable when applied to psyllid diversity, allowing biodiversity assessments [28], species identifications [43] and the description of new genera [17].

On the other hand, scarce information is available on the attraction of psyllids to light. Studies have been conducted mostly on the pest species *Diaphorina citri* Kuwayama, determining a positive phototactic behavior [44]. However, this appears to be limited only to UV and yellow and green colors [45]. In Australia, studies on color detection on psyllids have been undertaken on a few species of the family Aphalaridae, highlighting species-specific light sensitivities [46], with some species more attracted to yellow-green colors, and others to red stimuli [47]. Otherwise, the literature on psyllids light trapping is understandably scarce, with just a few records (targeting leafhoppers, planthoppers and psyllids conducted in Europe), showing generally low numbers of psyllids compared to many thousands of other insects collected [34,48], or a larger prevalence of a few taxa over the total number of specimens collected [37]

To date, no studies have yet been undertaken to assess the efficacy of light traps for the collection of psyllids in Australia. The aim of this study was to target psyllids over a series of light trapping sessions in Queensland National Parks, to (i) assess the number and diversity of psyllid species attracted to light, and to (ii) determine the validity of this technique to study the biodiversity of this group. Furthermore, pairing light trapping with COI barcoding, we aimed to (iii) generate barcode sequences for each species collected to provide an insight into psyllid biodiversity and contribute to generating a COI psyllid database for future identifications.

## 2. Materials and Methods 

Field collection was undertaken in Queensland, Australia, during the month of December 2019, using the collection permit of the Entomological Society of Queensland for Queensland parks WITK18701717-3 and forests WITF18701717 granted FM (member of the society). 

**Figure 1:**
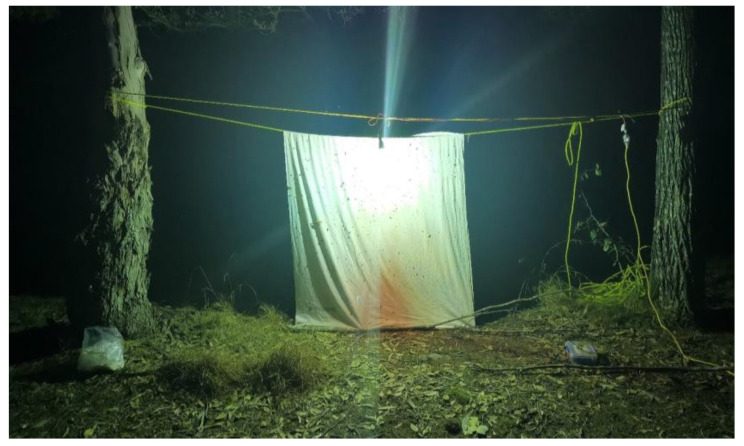
Light trap set during field collection.

A light trap was set up for eight nights for a period of 7 to 8 h, from 7 p.m. to 2/3 a.m., in different locations each night (Table 1, Table S1). A rope was straightened in the double passage between two trees, at a distance of 3–5 m from each other. A sheet was positioned on one of the lines, while a 160 Watts mercury vapor lamp powered by a generator (Honda 2KVA inverter model Eu20i) was fixed on the front line, being cautious not to touch the rope or the sheet behind (Figure 1).

**Figure 2:**
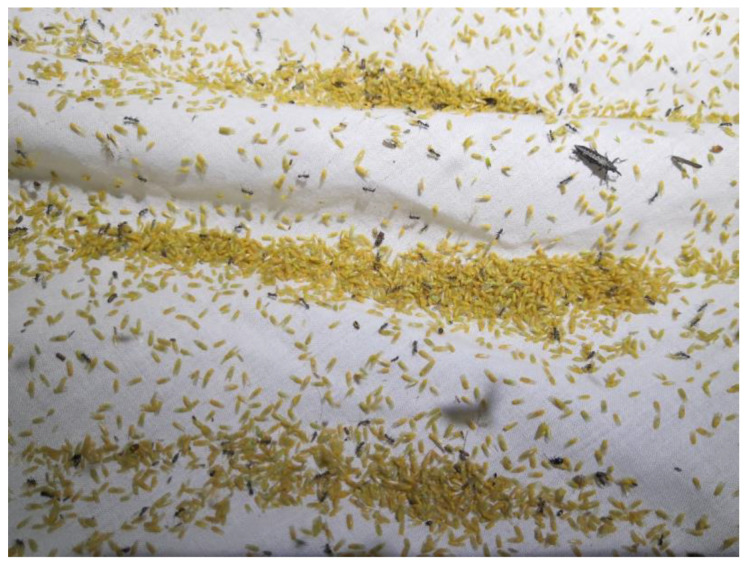
*Creiis* sp. “H” at light trap, Wide Water Reserve, near Taroom, Queensland (Photo by Peter Rühr).

Additionally, data were included here from a separate light trapping event, undertaken in Queensland by Erinn Fagan-Jeffries (University of Adelaide), James Dorey (Flinders University) and Peter Rühr (University of Cologne), in November 2019. The light trap design was based on that of Brehm [49], modified to run from 12V car outlet with the following specifications: 8× CUN66A1B near UV LED—365 nm, 4× Cree XP-E2 Royal Blau, 3× Cree XP-E2 Green, 1× Cree XP-G3 5500K. Psyllids belonging to a single taxon were recorded here in thousands; hence, this species was included in the analyses (Figure 2).

**Figure 3:**
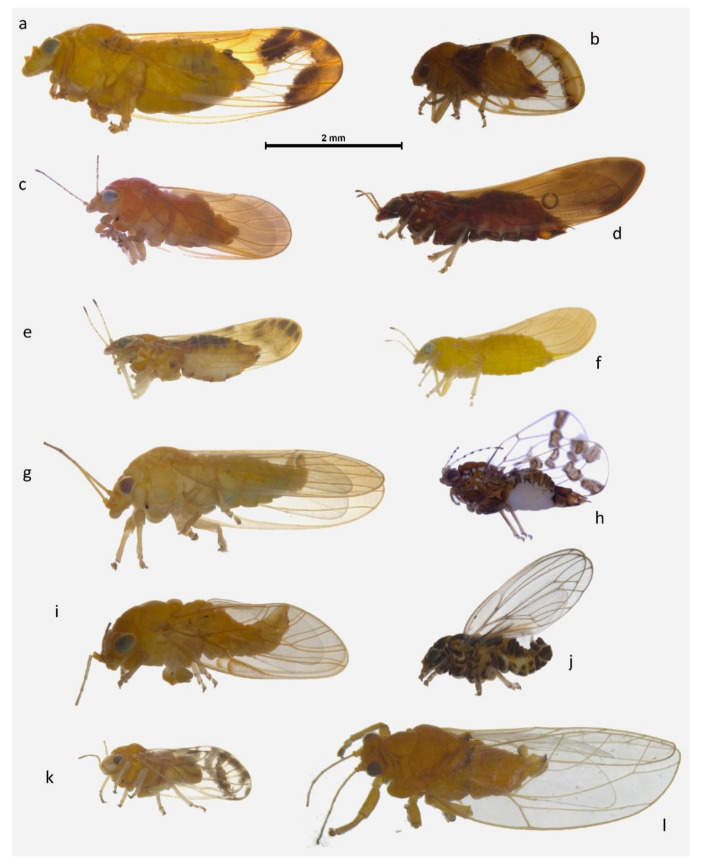
Examples of diversity in forms and colors of the psyllids collected. *Blepharocosta* sp. B ♀ (**a**), *Insnesia* sp. A ♀ (**b**), *Creiis* sp. L ♀ (**c**), *Anoeconeossa* sp. B ♀ (**d**), *Anoeconeossa* sp. D ♀ (**e**), *Anoeconeossa* sp. H ♀ (**f**), *Creiis* sp. J ♂ (**g**), *Pseudophacopteron* sp. A ♀ (**h**), *Phellopsylla* sp. C ♂ (**i**), *Phellopsylla* sp. I ♂ (**j**), *Acizzia* sp. A ♂ (**k**), *Schedotrioza* sp. A ♂ (**l**). Scale bar is 2 mm.

Psyllids were collected from the sheet by aspirating them using an entomological aspirator and preserved in high grade ethanol. In the laboratory, psyllids were morphologically examined and subdivided into morphospecies for each light trapping session and identified to the genus-level following available keys [3]. High-resolution auto montage photos of the psyllids were taken before DNA extraction using the Leica Application Suite software (version 4.5.0), from 10 to 20 stacked images obtained using a Leica stereo microscope M205C with a DFC450 camera. The software GNU Image Manipulation Program (GIMP) version 2.10.18 was used to collate some of these photos into a plate (Figure 3).

DNA were extracted from 93 individual psyllids representing the morphologically identified morphospecies. Non-destructive DNA extraction was performed using an overnight Proteinase-K digest of whole insects, following the protocol presented elsewhere for Muscidae [50]. 

A fragment of COI barcode region [41] of ~570 bp was targeted here using a newly designed primer, PsyCOI-F3 (5′-ACAATTGTTACWGCWCAYGC-3′), paired with the primer HCO2198 (5′-TAAACTTCAGGGTGACCAAAAAATCA-3′; [51]). The PsyCOI-F3 primer was designed from an alignment of *Diaphorina citri*, *Cacopsylla pyri*, and *Trioza adventicia*. The polymerase chain reaction (PCR) was performed using the MyFi kit (Bioline Meridian Biosciences, Cincinnati, USA) following the manufacturer’s instructions and the following cycle: initial denaturation at 95 °C for 2 min, followed by 40 cycles of 30 s at 94 °C, 45 s at 50 °C and 45 s at 72 °C, and a final elongation of 1 min at 72 °C. 

PCR products were Sanger sequenced in both directions by Macrogen Inc. (Macrogen, Seoul, Korea).

A second PCR was performed on three samples that did not amplify initially. This was performed using the same PCR kit and cycle, but with the primer pairs C1–J1709 (5′-AATTGGWGGWTTYGGAAAYTG-3′; [52])- HCO2198, and LCO1490 (5’-GCTCAACAAATCATAAAGATATTGG-3’; [51])- HCO2198. 

The electropherograms were manually examined and checked for pseudogenes and stop codons. Forward and reverse sequences were then paired using the software MEGA X [53]. An alignment was created using the same software and a pairwise distance matrix (Table S2) was generated using the Kimura-2-parameters model [54], which was visualized as a neighbor-joining tree to identify groups of taxa (Figure S1). Ultimately, each sequence was blasted against the online database GenBank and BOLD (Table S1), to assess its identity.

## 3. Results

During eight sessions of light trapping, a total of 667 (234 males and 433 females) individual psyllids were collected. After morphological examination, these were initially identified to 5 families, 22 genera and 79 morphospecies (Table 1). To these, a species from a separate collection event (Figure 2) was added, represented by hundreds of individuals that were not individually counted, bringing the total number of morphospecies to 80.

The DNA extractions, amplifications and sequencing were performed here on a total of 93 specimens, representing all morphospecies plus a few additional individuals considered of dubious identification. Ultimately, this work generated a total of 91 COI sequences of 533 bp, deposited on NCBI GenBank with accession numbers MT375232-MT375322.

Of the 91 sequences generated, 37 were grouped into 16 clusters in a pairwise distance matrix (Table S2), with a similarity to each other higher than 99%, suggesting they belonged to 16 different taxa. The remaining 54 sequences were more than 5% dissimilar to each other and to these 16 clusters. Therefore, the overall number of taxa recorded using COI barcoding was 70 species, belonging to 22 genera and 5 families (Table 1). Each COI sequence was blasted on GenBank and BOLD to ensure it was not the result of contamination and to compare it to other sequences present there. Of the 91 sequences blasted, only 9 had a similarity >99%, matching sequences were present on the GenBank database, while the remaining 82 showed very low similarities, usually between 80–90% (Table S1). Of the nine sequences that matched voucher sequences on the database, eight belonged to the described species while one matched an “*Australopsylla* sp.” Sequence.

The total number of taxa recorded in this study was 72, including 70 confirmed by the COI barcoding data and another two morphospecies which failed PCR amplification. Each light trapping event collected from a single taxon, up to 23 different taxa during the same night and from just two individuals, up to 197 (Table 1, without considering the separate event).

Of the 72 taxa recorded here, 62 belonged to the family Aphalaridae (15 genera), six to the Psyllidae (two genera), two to the Triozidae (two genera), and one each to Calophyidae and Phacopteronidae (Figure 3; Table 2; Table S1). A total of eight taxa were collected with more than 10 individuals and all belonged to the family Aphalaridae (genera *Creiis, Cryptoneossa, Ctenarytaina, Glycaspis* and *Phellopsylla*) (Table 3). Of these, three morphospecies were recorded in numbers higher than 100 individuals: *Phellospylla* sp. C, *Creiis* sp. H and *Cryptoneossa triangula* (Table 3).

## 4. Discussion

While light trapping is widely known to be a useful tool for insect collections and biodiversity assessments, scarce data was found in the literature about psyllids trapped using light sources. This might be due to the fact that, when sampling for psyllids, entomologists would generally prefer to collect them from their host plants, since species descriptions are greatly enhanced by valuable, and sometimes diagnostic, host plant information. Here, despite not being able to record host plant information for these psyllids, this study confirmed that light trapping can be considered a useful tool to assess the diversity of this group for a certain geographic area. Host plant information remains important and often a fundamental aid during species identification, as well as species description. For these reasons, we suggest that psyllid light trapping should be considered a valid tool for a first biodiversity assessment and that it should be followed up by host plant-targeted collections, to obtain more information on each psyllid species of interest.

The preliminary morphological examination highlighted the presence of 80 morphospecies (79 from the light trapping series and 1 from the separate event). On the other hand, the COI barcoding analysis confirmed the presence of only 70 taxa, based on a genetic variation >5%, which is even higher than the 3% variation usually considered a valid threshold for a species level separation in psyllids [17,28]. The reason of the discrepancy between morphological and genetic species assessment—beside the two species that failed PCR amplification—can be found in the fact some of the taxa were represented by a single (or few) specimen(s), belonging to a single sex. This initially led us to consider males and females of some species as two separate taxa, due to strong sexual dimorphism. This issue highlights the value of COI barcoding analysis in comparing genetic distances for species identifications, even when a single specimen was collected. Furthermore, the adoption of a non-destructive DNA extraction method allowed a second morphological examination of tentatively identified specimens, which confirmed the genetic species delimitation based on *a posteriori* taxonomic validation. 

This study recorded a total of 72 species from 22 genera and five families. Of these, 67 species are possibly undescribed. The use of DNA barcoding to obtain a COI sequence for each morphospecies contributed to confirm morphospecies diversity, even providing precise species identification in five cases, when a closely matching COI sequence belonging to a described species was available on an online database. In a single case (Sample 15; Table S1), the COI sequence matched a sequence belonging to an undescribed *Australopsylla* species (Table S1). Interestingly, the morphology of the specimen we collected—costal margin of wings bearing setae and vein Cu_1b_ not being strongly recurved—quite clearly identifies it as belonging to the genus *Blepharocosta* (sp. B; Figure 3a) and not *Australopsylla*, suggesting that this might be a case of incorrectly labelled COI sequence on GenBank. 

However, for the remaining 67 taxa, closely matching COI sequences were not available on GenBank or BOLD. This shows the lack of a comprehensive psyllid COI database present online and highlights the importance of generating sequences from both unidentified species and species with a confirmed taxonomical identification, as has recently been suggested elsewhere [55]. The need for a complete reference database is highlighted in our study, not only to better understand biodiversity, but also for biosecurity—two of the species we collected had their closest matches on GenBank with a major exotic pest, the Asian citrus psyllid *Diaphorina citri* (Table S1). Furthermore, of the few taxa identified to species level, two species are new records for the state of Queensland: *Cryptoneossa triangula* and *Trioza adventicia* [3,43].

Here, by generating 91 COI sequences belonging to 72 species, we provided a useful tool that will improve psyllid representation on the online database and allow comparisons for a number of genera previously not represented. Most importantly, pairing COI barcoding with light trapping provided important information, not only for the biodiversity of psyllids in this area of Queensland, but enabling a better understanding of the psyllid diversity of Australia. Of particular interest are the species belonging to the genera *Atmetocranium* (Calophyidae), *Insnesia* (Psyllidae) and *Pseuodophacopteron* (Phacopteronidae).

The genus *Atmetocranium,* temporarily placed in the family Calophyidae [56], is represented by a single described species, *Atmetocranium myersi* Tuthill 1952, native to New Zealand and reported in Australia in 2004 [3]. However, the species collected here, despite showing the typical lack of cranial suture characteristic of the genus, also appears to be morphologically very different in the shape of the wings from *A. myersi*, suggesting that it might be a second undescribed species belonging to the same genus. The genera *Insnesia* and *Pseudophacopteron* are both poorly studied and represented in Australia by undescribed taxa only [3]. In the case of *Pseudophacopteron,* this genus is the sole representative of its family in the country [3,57]. Therefore, the new records reported here provide additional information on the distribution and diversity, not only for individual taxa, but also for some of the most poorly studied genera and families of the Australian psyllid fauna. 

Furthermore, only five taxa were identified here to a species-level using COI barcoding, while another 67 taxa remained unidentified. This corresponds to a ratio of ~13:1 unidentified/identified species. While it was not possible to identify morphologically to a species-level the 67 taxa, it is safe to hypothesize a high number of these are undescribed. For example, the genera *Creiis* and *Phellopsylla* were thought to be represented by eight and six described species in Australia, respectively [3]. On the other hand, 15 taxa of *Creiis* and 11 of *Phellopsylla* were recovered in this study. Based on these results, we can hypothesize that the number of psyllid species present in the country is much more diverse than expected. This is especially interesting considering that the vast majority of species collected here belong to the family Aphalaridae, while high numbers of undescribed species had been previously recorded, mostly from the genus *Acizzia* (Psyllidae) [40]. Additionally, the results presented here were based on field collections performed in the sub-tropics of Eastern Australia, suggesting that an even higher taxonomic diversity could be recorded in areas of the Northern Australian tropics, which are known to harbor even higher diversity [58,59]. 

Based on these aspects and on the ratio of undescribed-to-described species recorded here, a conservative estimate of the number of undescribed psyllid species in Australia would at least equate to the number of described species reported by Hollis [3], bringing the total number of Australian psyllids up to 700–800, but it could be hypothesized to be many times higher, in the order of thousands psyllids species.

Some of these species were recorded in high (>10 individuals) or very high (>100s) numbers. These results suggest that some species of psyllid are intrinsically attracted to light sources. In particular, there was an exceptionally strong bias in high numbers belonging to the family Aphalaridae, with comparatively low attraction to light in the families Psyllidae (8 specimens out of ~700), Calophyidae (5 specimens), Triozidae (2 specimens) and Phacopteronidae (1 specimen). This is in stark contrast to other collection methods otherwise utilized in biodiversity studies, such as sweep-netting individual host plants: in the case of the Bush Blitz survey of the Great Victoria Desert, South Australia, for example, yielded 28 species of Psyllidae, eight species on Aphalaridae and seven species of Triozidae [60].

Here, of the Aphalaridae, the three species *Cryptoneossa triangula*, *Phellopsylla* sp. “C” and *Creiis* sp. “H” were recorded with more than 100 individuals during single trapping events. The species *Phellopsylla* sp. “C” was also reported in multiple locations (albeit at a lower density). Furthermore, the data recorded here reported a higher percentage of females compared to males, in a ratio that approaches 2:1. This shows an opposite trend compared to the results presented elsewhere for Hemiptera in general [34,48,61], where a prevailing number of males had previously been explained by differing sex-specific activities peaks at different times [61]. However, our result is in agreement with a previous work conducted specifically on psyllids, in the French city of Strasbourg [37]. Based on the high diversity of species collected both in that occasion (29 species) and in the present study, there are sufficient data to suggest this higher proportion of females attracted to light is the common pattern for psyllids.

## 5. Conclusions

This study suggests that psyllid light trapping can be a useful tool for the biodiversity assessments of poorly documented high-biodiversity areas. This methodology, when paired with COI barcoding, can provide precise information on the number of taxa present in a certain area, therefore enabling subsequent field collections targeting psyllids’ host plants. Furthermore, the application of this technique in the sub-tropical areas of Queensland allowed us to record the presence of undescribed psyllid species in very high numbers, suggesting that the Australian psyllid fauna is strongly understudied. Ultimately, based on the results obtained here, it appears that some psyllid taxa are more attracted to light than previously thought. Therefore, we encourage similar studies to be undertaken in other areas of Australia and the world, to enable a better understanding of the behavior of this group of ecologically important insects. 

## Figures and Tables

**Table 1:** Psyllids collected during each light trapping session. The table includes date, location, number of families, genera and individuals (N), collected divided by sex (♂, males; ♀, females). The number of species is reported based on the morphological assessment (Morph.), on the cytochrome oxidase c (COI) barcoding and on the joined total, including the 2 taxa that failed PCR amplification. The total number of families, genera and species is the overall number of different groups recorded across the eight sampling events (Table S1). Values marked with * have been estimated based on photos and not manually counted, therefore are excluded by the overall count.

Date	Location	Families	Genera	N	♂	♀	Morph/COI/Total
25 November 2019	Wide Water Reserve, Taroom	1	1	*	*	*	1/1/1
5 December 2019	Tambourine National Park	3	9	66	20	46	17/14/15
6 December 2019	Rainbow Beach–1	2	10	35	19	16	9/9/9
7 December 2019	Rainbow Beach–2	4	14	154	45	109	28/23/23
9 December 2019	Rockhampton	1	5	23	8	15	10/9/10
10 December 2019	Yeppoon	2	6	197	91	106	8/8/8
11 December 2019	Blackdown Tableland N.P.	2	9	186	51	135	17/16/16
12 December 2019	Cania National Park	1	3	3	0	3	3/3/3
14 December 2019	Inbil S.F.	1	1	2	0	2	1/1/1
		5	22	667	234	433	80/70/72

**Table 2:** Final psyllid sample identifications. Combined morphology and DNA barcoding results for 72 taxa collected. Additional details are in Table S1.

Family	Genus (# spp.)	Species	GenBank Accession
**Aphalaridae**	*Agelaeopsylla* (4)	*Agelaeopsylla* spp. A-D	MT375237-40
	*Anoeconeossa* (8)	*Anoeconeossa* spp. A-H	MT375241-50
	*Australopsylla* (1)	*Australopsylla* sp. A	MT375252
	*Blastopsylla* (1)	*Blastopsylla occidentalis*	MT375253-54
	*Blepharocosta* (2)	*Blepharocosta* spp. A-B	MT375255-58
	*Boreioglycaspis* (1)	*Boreioglycaspis melaleucae*	MT375291
	*Cardiaspina* (3)	*Cardiaspina* spp. A-C	MT375259-62
	*Creiis* (15)	*Creiis* spp. A-O	MT375263-79
	*Cryptoneossa* (1)	*Cryptoneossa triangula*	MT375280-81
	*Ctenarytaina* (2)	*Ctenarytaina longicauda*	MT375282-84
		*Ctenarytaina* sp. A	MT375285
	*Glycaspis (Glycaspis)* (4)	*Glycaspis* spp. A-D	MT375286-90
	*Lasiopsylla* (3)	*Lasiopsylla* spp. A-C	MT375293-96
	*Phellopsylla* (12)	*Phellopsylla* spp. A-L	MT375297-313
	*Phyllolyma* (3)	*Phyllolyma* spp. A-C	MT375314-17
	*Platyobria* (1)	*Platyobria* sp. A	MT375318
	*Spondyliaspis* (1)	*Spondyliaspis* sp. A	MT375321
**Calophyidae**	*Atmetocranium* (1)	*Atmetocranium* sp. A	MT375251
**Phacopteronidae**	*Pseudophacopteron* (1)	*Pseudophacopteron* sp. A	MT375319
**Psyllidae**	*Acizzia* (5)	*Acizzia* spp. A-E	MT375232-36
	*Insnesia* (1)	*Insnesia* sp. A	MT375292
**Triozidae**	*Schedotrioza* (1)	*Schedotrioza* sp. A	MT375320
	*Trioza* (1)	*Trioza adventicia*	MT375322

**Table 3:** Psyllid species collected with more than 10 individuals. The table includes date, location and data for each psyllid species collected with more than 10 individuals: family, species and number of males and females are reported.

Date	Location	Family	Species	N	♂	♀
25 November 2019	Wide Water Reserve, Taroom	Aphalaridae	*Creiis* sp. H	400+	200+	200+
5 December 2019	Tamborine National Park	Aphalaridae	*Ctenarytaina longicauda*	26	9	17
6 December 2019	Rainbow Beach -1	Aphalaridae	*Boreioglycaspis melaleucae*	13	9	4
6 December 2019	Rainbow Beach -1	Aphalaridae	*Ctenarytaina longicauda*	14	8	6
7 December 2019	Rainbow Beach -2	Aphalaridae	*Ctenarytaina sp.*	71	22	49
10 December 2019	Yeppoon	Aphalaridae	*Phellopsylla* sp. C	179	87	92
11 December 2019	Blackdown Tableland N.P.	Aphalaridae	*Phellopsylla* sp. K	23	11	12
11 December 2019	Blackdown Tableland N.P.	Aphalaridae	*Cryptoneossa triangula*	134	28	106

## Supplementary Materials

The following are available online at https://www.mdpi.com/2075-4450/11/6/354/s1: Table S1: Samples used in this study. The table lists the samples collected for this study divided by morphospecies. It includes the reference identification; the date, country, location and GPS coordinates of the sampling localities; the morphological identifications; the number of individuals collected for each sex; the match obtained when comparing the sequences obtained with GenBank and BOLD reference sequences (and the % of similarity with the closest match); and the final identification obtained, with the GenBank accession numbers associated with each sequence generated in this study. Table S2: Pairwise distance matrix. The table shows the pairwise distance matrix obtained using MEGA X and the K2P model. Figure S1: Neighbor Joining tree of the 91 COI sequences obtained in this study. The gene tree was generated using MEGA X, 10000 replicates, K2P model.
